# The Role of Geomagnetic Cues in Green Turtle Open Sea Navigation

**DOI:** 10.1371/journal.pone.0026672

**Published:** 2011-10-26

**Authors:** Simon Benhamou, Joël Sudre, Jérome Bourjea, Stéphane Ciccione, Angelo De Santis, Paolo Luschi

**Affiliations:** 1 CEFE, CNRS, Montpellier, France; 2 LEGOS, CNRS, Toulouse, France; 3 IFREMER, La Réunion, France; 4 Kélonia, La Réunion, France; 5 Istituto Nazionale di Geofisica e Vulcanologia, Roma, Italy; 6 Dipartimento di Biologia, Università di Pisa, Italy; University of California, Berkeley, United States of America

## Abstract

**Background:**

Laboratory and field experiments have provided evidence that sea turtles use geomagnetic cues to navigate in the open sea. For instance, green turtles (*Chelonia mydas*) displaced 100 km away from their nesting site were impaired in returning home when carrying a strong magnet glued on the head. However, the actual role of geomagnetic cues remains unclear, since magnetically treated green turtles can perform large scale (>2000 km) post-nesting migrations no differently from controls.

**Methodology/Principal Findings:**

In the present homing experiment, 24 green turtles were displaced 200 km away from their nesting site on an oceanic island, and tracked, for the first time in this type of experiment, with Global Positioning System (GPS), which is able to provide much more frequent and accurate locations than previously used tracking methods. Eight turtles were magnetically treated for 24–48 h on the nesting beach prior to displacement, and another eight turtles had a magnet glued on the head at the release site. The last eight turtles were used as controls. Detailed analyses of water masses-related (i.e., current-corrected) homing paths showed that magnetically treated turtles were able to navigate toward their nesting site as efficiently as controls, but those carrying magnets were significantly impaired once they arrived within 50 km of home.

**Conclusions/Significance:**

While green turtles do not seem to need geomagnetic cues to navigate far from the goal, these cues become necessary when turtles get closer to home. As the very last part of the homing trip (within a few kilometers of home) likely depends on non-magnetic cues, our results suggest that magnetic cues play a key role in sea turtle navigation at an intermediate scale by bridging the gap between large and small scale navigational processes, which both appear to depend on non-magnetic cues.

## Introduction

As the geomagnetic field is present everywhere at the Earth surface, it has been considered a major candidate for providing large scale locational cues, beyond its well-documented role in providing directional cues (geomagnetic compass; e.g. see [1]). Large scale oceanic travelers such as pelagic birds and sea turtles are likely to rely on geomagnetic locational cues because they have to navigate through vast stretches of featureless open sea where other cues may be unavailable. However, experiments involving pelagic birds [2], [3] showed that their navigational skills were not impaired when these birds were prevented from perceiving the geomagnetic field by carrying strong magnets on the head. In contrast, a number of experiments with hatchling and juvenile sea turtles in arenas showed that these animals are able to obtain locational information from the geomagnetic field [4].

In a previous homing experiment, we showed that geomagnetic information may help adult female green turtles (*Chelonia mydas*) to return to their egg-laying sites on an island after having been experimentally displaced in the open sea [5]. Some individuals were prevented from perceiving the geomagnetic field by carrying an extremely strong magnet glued on the head, either during the homing phase or during the displacement from the nesting beach to the release site. The turtles of both groups were nevertheless able to home, albeit less efficiently than controls. The impairment of the turtles of the former group provided evidence that sea turtles use geomagnetic cues to improve their pelagic navigation efficiency. The effect on the turtles of the latter group, whose magnet was removed just before release, could be explained in two ways: (i) the strong magnet produced some long lasting after-effect, which may cause a kind of “memory reset” of the geomagnetic location of the nesting site or (ii) the treatment prevented turtles from acquiring some critical route-based navigational information during the (passive) outward journey. More generally, it cannot be excluded that the application of a strong magnetic field to the turtles' head might result in poor navigation abilities because of some unspecific effects of the artificial magnetic field on brain functioning.

In the present study, we further investigate the role of geomagnetic information in green turtle open sea navigation in two ways. We studied the homing performances of nesting green turtles released in the open sea (1) when carrying a weak magnet on the head during the return path or (2) when wearing a very strong magnet (the same type as the one used in our previous experiment [5]) while still on the nesting beach. The field generated by the weak magnet had an intensity of the same order of magnitude as that of the Earth's magnetic field and so should make turtles experience an altered magnetic field providing biologically plausible but misleading magnetic cues. The treatment with the strong magnet applied before the displacement to the release point, aimed to test a possible long-lasting effect of strong magnets on the turtle navigational skills.

Thanks to the high spatial and temporal resolution provided by GPS tracking, we were able to analyze the turtles' navigational performances in detail, distinguishing different – initial, central and final – phases of the pelagic trips. These three phases are expected to involve a different balance between movement persistence and goal attractiveness, with the consequence that, from a practical point of view, the navigational efficiency during each phase has to be evaluated in a specific way (see Material and Methods). The central and final phases are also expected to involve different, scale-dependent navigation processes with different spatial resolutions, because of a trade-off between working scale and accuracy. Indeed, the navigational processes working at a large scale usually only enable an animal to reach a general area surrounding the goal location, whereas those allowing the animal to pinpoint the goal can work only at a small scale, when the animal is in the close vicinity of its target [4], [6]–[7]. The hierarchy of the navigational process required to reach a goal from a very distant starting point should therefore involve a series of concentric “circles of confusion”, each corresponding to a scale-specific navigational process. Each of these circles is centered at the goal and encompasses the set of locations that are indiscernible from the goal in terms of the cues used by the navigational process in question [8], [9]. Thus, when a homing turtle is within a few kilometers of its nesting site, it is likely to be within the circles of confusion of the navigation processes it used at larger scales. To reach its nesting site, it then should rely on a very small scale (pinpointing) process, for which an involvement of wind-borne (presumably olfactory) cues and/or visual cues of the goal, has been proposed [10], [11].

The homing tracks we recorded in previous studies [5], [12] showed that homing turtles, and particularly those that were magnetically treated, were usually able to navigate quite efficiency towards their nesting site but may miss it by a few dozen of kilometers (a result also confirmed in the present study). This suggested the existence of a medium scale, magnetic-based navigational process, enabling turtles to bridge the large scale (true pelagic) and the small scale (pinpointing) navigational processes. The distinction between the different phases of homing paths thus enabled us to examine at which specific spatial scale magnetic cues may play a major role during the sea turtle oceanic navigation.

## Results

**Figure 1:**
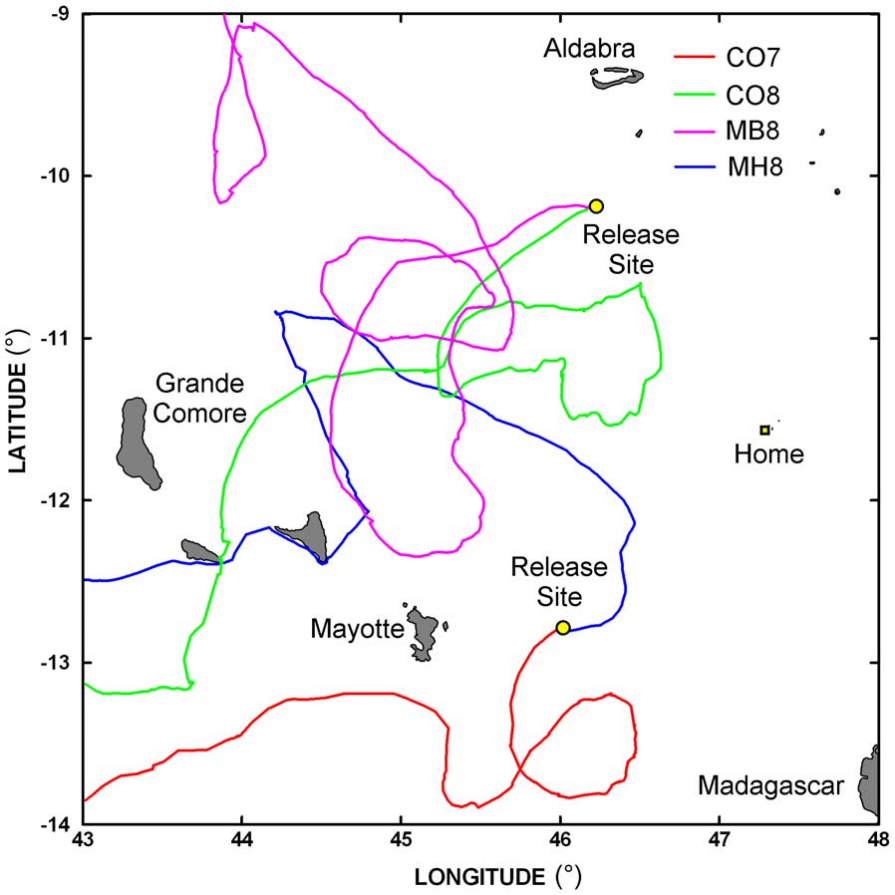
Paths of four turtles (two belonging to the CO group, one to the MB group and one to the MH group) that did not attempt to home but migrated towards their feeding sites along the African coast.

Eight out of the 24 GPS-tracked female turtles were magnetically treated for 24–48 h on the nesting beach prior to displacement (MB group) using a strong magnet, and other eight turtles had a weak magnet glued on the head at the release site (MH group). The last eight turtles were used as controls (CO group). Four turtles were removed from analysis because they did not show a high motivation to home (Fig. 1). Three of them (CO7, MB7 and MH7) moved more or less directly towards their feeding grounds along the African coast. A fourth one (CO8) initially orientated towards home, but she was only able to come only within 84 km of home before eventually giving up and moving towards her feeding grounds. The computation of the motor (water masses related; see Material and Methods) paths of these four females confirmed that their current-corrected headings were not consistently directed towards their nesting beach.

**Figure 2:**
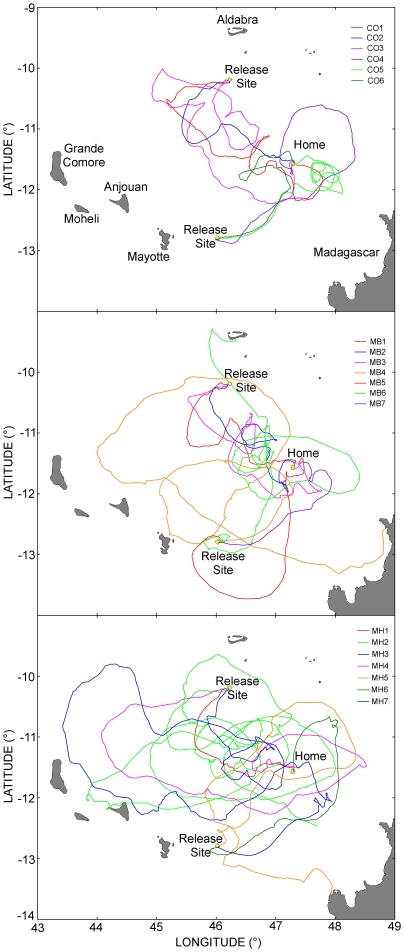
Paths of the 20 turtles which attempted to home, 16 of them being successful. The paths turtles belonging to the CO, MB and MH groups are represented in the top, middle, and bottom panel respectively.

**Figure 3:**
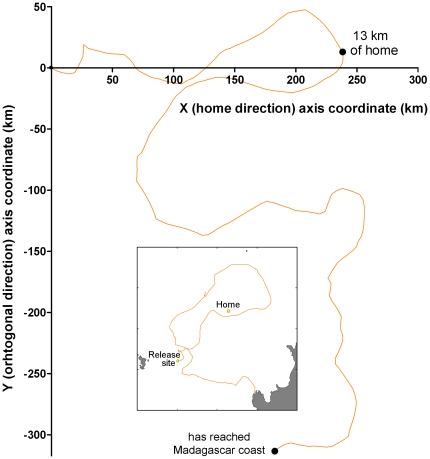
Example of motor (i.e. current corrected) path of a MH turtle (MH5), which was able to come close to home (13 km) in four days but missed it and was eventually unable to reach it. In the special frame of reference used here, the X axis corresponds to the home direction, and the Y axis to the orthogonal direction (*X_k_* = *X_k−1_*+*l*.cos(θ*_k_*−γ*_k_*_−1_); *Y_k_* = *Y_k−1_*+*l*.sin(θ*_k_*−γ*_k_*_−1_), where *l* = 5 km is the step length, θ*_k_* is the orientation of the *k* ^th^ step, and γ*_k_*_−1_ is the goal direction at the *k*−1^th^ location). The inset shows the ground-related path in the geographic frame of reference. It can be clearly seen that this magnetically treated turtle was quite efficient in moving in the home direction during the first part of its homing path: to come within 13 km of home, she swan only 305 km (61 5-km steps) for a move of 239 km in the goal direction (navigational efficiency: 0.78).

Three of the remaining 20 turtles (MB4, MB6, and MH5) were also unable to home. They covered long distances often along convoluted routes while apparently searching for home (Fig. 2), and eventually abandoned homing by stopping at another place (Aldabra Island for MB6, Madagascar for MB4 and MH5). They nevertheless showed a strong motivation to home and were able to arrive a few (14–27) kilometers of home during their quests. Indeed, their motor paths were globally oriented homewards until they were close to home (Fig. 3). This also applied to a fourth turtle (MH2) whose Argos/GPS device stopped working after 54 days (probably due to exhausted batteries), while the turtle was still searching for home (so we do not know if this turtle eventually did or did not home). The movements of these four turtles were therefore analyzed exactly in the same way as those of the 16 successful ones.

Track durations and lengths (calculated from release point to home or, for non-homers, to the point they abandoned homing) suggest that CO and MB turtles behaved similarly, and that MH turtles were partially impaired (Table 1, columns 2 and 3). However, from a detailed examination of the whole set of tracks (Fig. 2), it is quite clear that MH turtles may have initially navigated similarly to CO and MB turtles but started to become impaired when arriving relatively close to home. The simple computation of the mean path lengths required to halve the distance to home confirmed this impression. These mean (±SE) lengths were 203±42 km in the CO group, 243±41 km in the MB group, and 269±65 km in the MH group, while the mean (±SE) lengths of the paths required to complete homing (or abandon) were 518±116 km in the CO group, 726±273 km in the MB group, and 1090±418 km in the MH group.

**Table 1:** Turtles' homing performances.

Turtle	Homing duration	Homing length	Global efficiency	Initial phase	Central phase	Final phase
CO1	13 days	806 km	0.75	18	0.84	2032 km^2^
CO2	6 days	447 km	0.72	12	0.80	810 km^2^
CO3	16 days	1132 km	0.58	0	0.56	648 km^2^
CO4	10 days	682 km	0.27	0	0.24	769 km^2^
CO5	9 days	786 km	0.71	0	0.72	865 km^2^
CO6	6 days	472 km	0.50	0	0.41	3335 km^2^
**mean±SE**	**10±2**	**721±103**	**0.59±0.07**	**5.0±3.3**	**0.60±0.10**	**1410±438**
MB1	6 days	484 km	0.63	11	0.68	710 km^2^
MB2	6 days	641 km	0.69	4	0.83	1289 km^2^
MB3	15 days	806 km	0.67	6	0.76	2264 km^2^
MB4^a^	*29 days*	*2122 km*	0.28	0	0.69	43241 km^2^
MB5	6 days	593 km	0.29	52	0.97	598 km^2^
MB6^b^	*29 days*	*1746 km*	0.36	21	0.76	14688 km^2^
MB7	5 days	395 km	0.30	0	0.33	1033 km^2^
**mean±SE**	**14±4**	**970±257**	**0.46±0.07**	**13.4±7.0**	**0.72±0.07**	**9118±6001**
MH1	6 days	368 km	0.80	3	0.78	841 km^2^
MH2^c^	*54 days*	*3646 km*	0.47	15	0.58	37399 km^2^
MH3	31 days	1870 km	0.55	0	0.80	77896 km^2^
MH4	23 days	1255 km	0.31	40	0.71	4563 km^2^
MH5^d^	*18 days*	*1265 km*	0.17	0	0.80	18783 km^2^
MH6	9 days	643 km	0.29	7	0.23	867 km^2^
MH7	6 days	463 km	0.42	0	0.44	911 km^2^
**mean±SE**	**21±7**	**1359±431**	**0.43±0.08**	**9.3±5.5**	**0.62±0.08**	**20180±10902**

a came only within 23 km of home in 60 hours; path stopped at 203 km of home (Madagascar).

b came only within 27 km of home in 14 days; path stopped at 256 km of home (Aldabra).

c came within 21 km of home in 44 days; path stopped at 112 km of home (battery exhausted).

d came only within 13 km of home in 4 days; path stopped at 227 km of home (Madagascar).

The global path and central phase efficiencies were estimated as the mean cosine of directional errors. The initial phase efficiency was estimated as the number of 5-km steps travelled (with respect to water masses) before the turtle considered definitely took the correct ±90° direction. The final phase efficiency was estimated as the mean of the squared distances between successive locations and home when the turtle came within 50 km of home. CO: control group; MB: magnetic treatment on the nesting beach, prior to displacement; MH: magnetic treatment during homing.

However, variables such as homing duration or track length are too coarse to provide reliable figures of homing efficiency because they are quite sensitive to the drift of oceanic currents (see Material and Methods). The current speed (with respect to ground) and the turtles' swimming speed (with respect to water masses) can indeed be very similar (a few kilometers per hour), so that the resultant track durations and lengths could be dramatically affected by the direction of the currents encountered. The turtles' motor (water masses-related) movements better represent the turtles' orientation behavior than their recorded, ground-related, movements [5], [12]. The global analysis of motor paths, as well as the analyses of initial and central phases of these paths (Table 1, columns 4–6), did not show any significant difference in homing efficiency between CO and MB or MH turtles. Both magnetically-treated groups performed worse than CO turtles, although not significantly, either globally or during the initial phase (Table 1, columns 4 and 5), but their mean efficiency was similar to that of CO turtles during the central phase (Table 1, column 6). It is worth noting that about half of the turtles of each group were able to move in the correct hemicycle (home direction ±90°) at the release site (0 values in column 5 of Table 1), suggesting that turtles chose their initial moving direction (first 5-km step) at random, independently of the treatment they had been subjected to. During the final phase (Table 1, last column), MH turtles significantly performed less efficiently than CO turtles (exact permutation test: p<0.03). MB turtles also seemed to perform less efficiently than CO turtles, but the difference was not statistically significant (p>0.10).

It is also worth noting that many turtles, irrespective of the group to which they belonged and the release site, showed a tendency to initially move in a common direction that was different from the home direction. They thus made a sort of mistake in their orientation over the first few days. This was especially clear in both 2008 releases (north-western site), when all turtles initially moved roughly south-westwards, and in the first 2009 release (south-western site), when 5 out of the 6 released turtles (2 CO, 2 MB and 1 MH) moved first eastwards before shifting north-eastwards (Fig. 2). This initial bias was only partly due to the action of currents, as such a tendency to display a common biased orientation is evident in the current-corrected motor paths as well.

## Discussion

Our results show that turtles exposed to a strong magnetic field for one or two days at the nesting site prior to displacement (MB group) or carrying a weak magnet on the head during the homing trip (MH group) were not particularly impaired with respect to controls before they arrived within 50 km of home. The mean homing performance of the turtles belonging to the MB or MH groups appeared to be lower than that of the control group once they arrived within 50 km of home, but the difference was statistically significant only for the MH group. The hypothesis of a long-lasting after-effect exerted by strong magnets [5], which might have cause a kind of “memory reset” of the geomagnetic location of the nesting site, is therefore not supported. However, it cannot be excluded that the absence of statistical significance for the MB group may have been due to a lack of statistical power caused by the small samples of the present study. Further investigations thus would be necessary before reaching a definitive conclusion. More importantly, it clearly appears that a homing green turtle does not need access to geomagnetic information when navigating far from its goal. This result is in general agreement with previous findings by Papi et al. [13], who showed that magnetically-treated green turtles were not impaired during their oceanic (trans-Atlantic) migration from their nesting site at Ascension Island to their Brazilian feeding grounds (more than 2000 km westwards). In contrast, MH turtles appeared to be dramatically impaired once they arrived relatively close to their goal. This suggests that geomagnetic cues would be really useful to navigating turtles only at this late stage.

**Figure 4:**
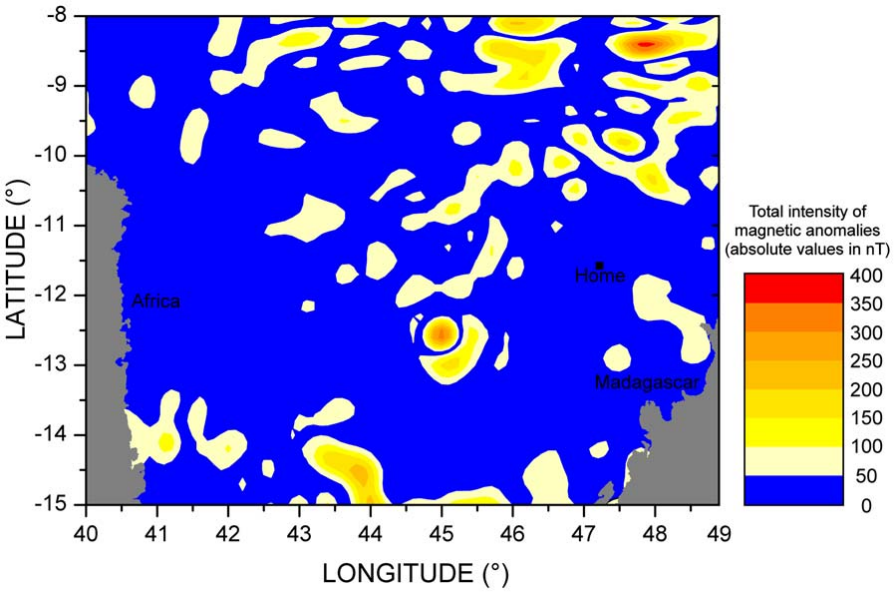
Map of geomagnetic anomalies around Glorieuses Islands (Home). This map has been constructed as the absolute value of the difference in the total intensity between Enhanced Magnetic Model 2010 and World Magnetic Model 2010 (see www.ngdc.noaa.gov/geomag/). The former is a complete representation of the real magnetic field up to a spherical harmonic degree n = 720 (minimum wavelength L = 40000/n = 56 km, corresponding to a spatial accuracy of 28 km). The latter corresponds to the main (outer core) field model. The difference between the two models is a good representation of the crustal magnetic field.

The picture emerging from our results is that green turtles would rely on non-magnetic cues (whose actual nature remains to be determined) to navigate at large scale through the open sea, shift to magnetic ones when closer to their target, and shift again to non-magnetic cues for the very final, pinpointing stage [4]. A possible reason why sea turtles would not rely on geomagnetic cues to estimate the goal direction at large distances is that there exist numerous magnetic anomalies in the open sea [14], [15]. A number of magnetic anomalies with intensities above 50 nT could be identified in our study area (Fig. 4). These anomalies appear to be strong enough to prevent the use of geomagnetic cues in a large scale (hundreds of kilometers) navigational system. Indeed, most green turtles nesting on islands in the Northern Mozambique channel have their feeding grounds along the African coast, about 1000 km westwards (unpublished data). In this part of the world, the geomagnetic intensity globally changes by about 1.1 nT/km along the migration route. Such a situation is not limited to our study area but seems to be quite widespread. For instance, the change is about 1.5 nT/km along the migration route of green turtles nesting on Ascension Island. Under such circumstances, if green turtles would rely on geomagnetic cues to perform their large scale navigation, even the weakest anomalies they cross would involve large localization errors (several dozen of kilometers), and turtles could also be easily “trapped” in wrong places characterized by a magnetic intensity close to the one experienced at destination. Consequently, it would be a much safer option for sea turtles migrating from their foraging grounds to their nesting sites or *vice versa* to rely on a non-magnetic navigational process until they arrive relatively close to their goal.

As the oceanic environment is apparently featureless, the pelagic navigation process should be based on large scale environmental gradients, possibly of olfactory nature as proposed for homing pigeons [16]. The initial directional bias affecting most turtles, which was also observed in our previous experiment [5], suggests that this large scale navigation process rests on a mixed “getting-there” – “knowing-where” solution. The fact that sea turtles are not able to compensate for the current drift, although they are able to correct it, leads to the same conclusion [12]. A pure “getting-there” solution involves a mechanical procedure enabling an animal to reach its goal without any locational knowledge (e.g. gradient following), whereas a pure “knowing where” solution involves some kind of cognitive map. Numerous navigational processes appear to mix elements from these two types of solutions, involving the joint use of mechanical procedures and partial spatial memory [7]. In the present case, the biased initial orientation may be due to the reliance of the turtles on non-orthogonal gradient fields considered independently from each other [17]. Furthermore, the fact that this bias was shown by turtles from all three groups in a similar way provides additional indications that this large scale pelagic process rests on non-magnetic information.

This non-magnetic process operating at large scale is likely to be imprecise, i.e. characterized by a large circle of confusion, within which turtles may then shift to another navigational process based on local geomagnetic cues to approach further their nesting site. Geomagnetic cues may indeed be used by green turtles a few dozen of kilometers around home, as indicated by the present findings because, even in the presence of anomalies, the geomagnetic field should remain sufficiently monotonical (i.e. predictable) at this smaller scale to allow navigation based on its local characteristics. To use such a navigational process, turtles would need to memorize the local characteristics of the geomagnetic field around the home location (which may be quite different of the global characteristics expected at larger scale because of the presence of an anomaly). This may be achieved through some kind of learning taking place during their previous visits to the home area. As sea turtles tend to be faithful to their place of birth and use it later as nesting site [18]–[20], this learning may at first rest on some form of geomagnetic imprinting [21], and would be regularly reinforced and updated later in life at each every new breeding season (every 3–4 years for female green turtles in our study area [22], and possibly more often for males [23], which hence might have improved island finding abilities than females). Like for the large scale non-magnetic process, this medium scale magnetic process may rely on a mixed “getting-there” – “knowing-where” procedure, possibly involving local gradients of total intensity and inclination [4].

Under this scenario, the sea turtle long-distance navigation in oceanic environments would be based on three successive navigational processes: 1. A large scale, non-magnetic process to reach the relative vicinity of the target; 2. A medium scale magnetic process, to be used when approaching the circle of confusion of the large scale non-magnetic process, based upon predictable magnetic gradient fields around the nesting area, whose characteristics are learnt (and updated) during successive visits; 3. A third, small scale, pinpointing process based on non-magnetic cues (presumably wind borne and/or visual cues; [4]), to be used when approaching the circle of confusion of the magnetic process. By acting at the intermediate scale, the magnetic process would play a key role in green turtles by bridging the gap between large scale and small scale, both non-magnetic, navigational processes. This scenario is based on results obtained on relatively small samples (for logistical reasons, it is always hard to work with large samples in this kind of experiment). Further experiments will therefore be necessary to confirm our results. In particular, it is quite possible that the absence of significant difference during the final homing phase between turtles exposed to a strong magnetic field before displacement and controls derived from the low statistical power inherent to small samples. We could not exclude that the significant difference during the final homing phase between controls and turtles equipped with a weak magnet during the whole homing phase was due to the behavior of some particularly unlucky individual belonging to the magnetic group. This seems however unlikely because most turtles in this magnetic group did appear to be disturbed during the final homing phase, only a few ones appearing lucky enough to quickly reach their home. Furthermore, despite the smallness of the samples, the navigational efficiencies of the three groups during the central (i.e. pelagic) phase are sufficiently consistent within and across groups to enable us to claim with confidence that a magnetic perturbation has no significant effect on the turtles' navigation behavior during this phase.

## Materials and Methods

### Subjects and experimental treatments

The experiment was performed in accordance with institutional and national (French) guidelines and regulations (Permit number 34-100, covering any behavioral experiment conducted on vertebrates in the wild, including the present one, attributed to the senior author and approved by Veterinary Services of the French Ministry of Agriculture).

A total of 24 female green turtles served as subjects. They were caught during the night at their nesting site on Grande Glorieuse (11.57°S, 47.29°E), a small, isolated island in the northern part of the Mozambique Channel. They were then kept on the beach in wooden crates for 24 to 48 h. Crates were placed in the shade and turtles were regularly wetted with seawater during the day to minimize their stress. Six turtles, two of each group (see below), were displaced at the same time using an aluminum (amagnetic) boat. They were released in the open sea 190–200 km from their nesting site. Two north-western displacements were performed in May 2008 (release site coordinates: 10.19°S, 46.23°E) and two south-western displacements in June 2009 (release site coordinates: 12.79°S, 46.02°E). During the boat travel, which lasted around 24 h, the turtles were kept in covered wooden crates to prevent them from seeing the sky and to protect them from the sun. They were also regularly wetted with seawater.

**Figure 5:**
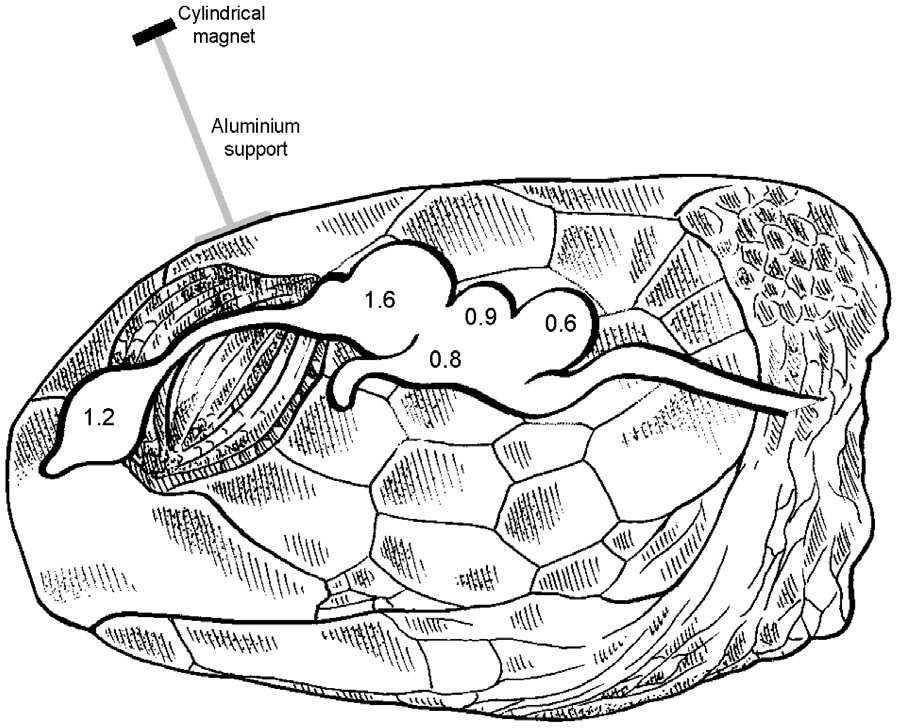
Intensity *B* of the magnetic field, expressed in µT, induced in various parts of a green turtle's brain by a cylindrical magnet placed horizontally 6.5 cm above the head. The values were computed as *B* = 0.1 *m* (3cos^2^(δ)+1)^0.5^/*d* ^3^ where *m* = 0.015 A.m^2^ is the magnetic moment of the cylindrical magnet, *d* is the distance from the magnet expressed in meters, and δ is the angular deviation from the cylinder axis (colatitude). The drawing of the turtle's head and brain was adapted from Fig. 172 in [25].

In our study area, the total intensity of the geomagnetic field is about 34 µT, and it changes by about 1.8 nT/km along a WSW-ENE axis. The expected (i.e. without taking anomalies into account) difference between home and NW and SW release sites were 200 and 350 nT, respectively (www.ngdc.noaa.gov/geomagmodels/struts/calcIGRFWMM). Turtles were assigned to three groups of eight: two experimental groups – Magnetic Beach (MB) and Magnetic Homing (MH) – and a control group (CO, no treatment except displacement). Turtles belonging to the MH group were magnetically treated during the homing trip by putting a weak magnet above their heads just before release. We used a 5 mm long cylindrical magnet, with a very small magnetic moment (*m* = 0.015 A.m^2^) that we placed horizontally 6.5 cm above the head using an aluminum inverse-T-shaped support. Given that the exact location of the biological magnetoreceptor is not known in turtles (as well as in any animal; [24]), increasing the distance between the magnet and the head enabled us to minimize the variations of the total intensity of the artificial magnetic field induced across the brain (Fig. 5). The total intensity of the resultant magnetic field (vectorial sum of the geomagnetic field and the magnetic field induced by the weak magnet) experienced by MH turtles in any part of their brain thus remained within the range of the geomagnetic field, but corresponded to intensities experienced at locations several hundreds of kilometers away from the actual location. Turtles belonging to the MB group were magnetically treated by gluing a very strong magnet (*m* = 1.2 A.m^2^) to the top of the head, but only while they stayed in wooden crates on the beach. These strong magnets, identical to those used in previous experiments [5], [13], generated a magnetic field larger than 500 µT across the whole brain. They were removed as soon as the boat left Grande Glorieuse (treatment time: 24–48 h).

Because the possible impairment due to wearing a very strong magnet at the nesting site before displacement and to wearing a weak magnet during the homing trip are likely to be qualitatively different, we thought that the quantitative comparison of the homing performances of MB and MH groups was not meaningful. We therefore considered that we performed a two-in-one experiment, with a common control group, rather than a single three- group experiment. Statistical comparisons of the turtles' navigation performances were therefore performed between CO and MB turtles on one hand, and between CO and MH turtles on the other hand, using exact permutation tests, which are the most powerful tests that can be performed when sample sizes are small.

### Movement recordings and oceanic current corrections

The turtles' movements were recorded with MK10 Argos-linked GPS loggers (Wildlife Computers, Seattle WA). These devices can acquire GPS locations through ‘Fastloc™’ technology during turtle surfacings, store them in an onboard memory and transmit them to the Argos system satellites. The GPS locations were programmed to be acquired every 20 min, but only a fraction of the programmed locations was eventually acquired and stored, and only a fraction of the stored locations could be transmitted (probably because of the low bandwidth and intermittent satellite coverage of the Argos system). We eventually obtained about one GPS location per hour, which allowed us to reconstruct the homing journeys with fair accuracy.

Green turtles' oceanic movements take place in the upper layers of the water column (10–20 m depth; [26]) and so are affected by surface currents. The recorded homing movements therefore corresponded to the vectorial sum of the turtles' own ‘motor’ movements within the water masses and the action of surface currents. In the Mozambique Channel, oceanic currents are far from being negligible: their speed can be of the same order of magnitude as of a turtle's speed within the water masses. Thus, a turtle may even actually move away from home while it is swimming homewards. As we showed in two previous studies [5], [12], green turtles are not able to compensate for the current drift, although they are able to correct it: they are indeed able to continuously update the home direction after displacement due to current drift (as well as after the passive displacement by boat), but are unable to adopt a voluntary biased heading to anticipate the current drift. To reliably estimate the turtles' navigational efficiency, we therefore estimated their motor (current-corrected) movements, which better represent the turtles' orientation behavior than the recorded, ground-related movements.

Surface current velocity fields were computed as the vectorial sum of geostrophic and Ekman components [27]. The geostrophic component results from the balance between the horizontal pressure gradient force and the Coriolis force. It was computed as the vectorial sum of the mean geostrophic surface currents, calculated from the mean dynamic topography, and the currents due to geostrophic anomalies, derived from the Ssalto/Duacs gridded altimetric Sea Level Anomaly data available weekly on a 1/3° grid (www.jason.oceanobs.com). Note that the geostrophic component was computed using an updated model, based on a new assessment of the mean dynamic topography [28]. The Ekman component results from the balance between friction by wind and the Coriolis force. It was estimated from daily wind stress data provided by Quikscat scatterometry on a 1/2° grid (www.ifremer.fr/cersat). Both components underwent a bi-linear spatial interpolation so as to get 1/4° velocity fields, and then the geostrophic component underwent a temporal third-order Lagrange polynomials interpolation [29] to obtain both geostrophic and Ekman fields on a daily basis. The two fields were then vectorially summed up to obtain the global surface velocity field at 1/4° on a daily basis. The oceanic current velocity occurring at each turtle location was then estimated through spatial and temporal interpolation from the daily global maps. By applying it to Argos-tracked drifting buoys (whose movements were only due to currents), this method was shown to provide reliable estimates of mesoscale current velocities [27], except for coastal locations. Daily surface current velocity maps at 1/4° resolution, as well as a user-friendly program making it possible to easily compute the oceanic current velocities at specified locations worldwide, can be downloaded from www.legos.obs-mip.fr/contacts/page-perso-equipe-dynbio/joel-sudre. The oceanic current velocity occurring at any turtle's location was then subtracted from the turtle ground velocity at this location to obtain the turtle motor velocity (see [12] for details).

**Figure 6:**
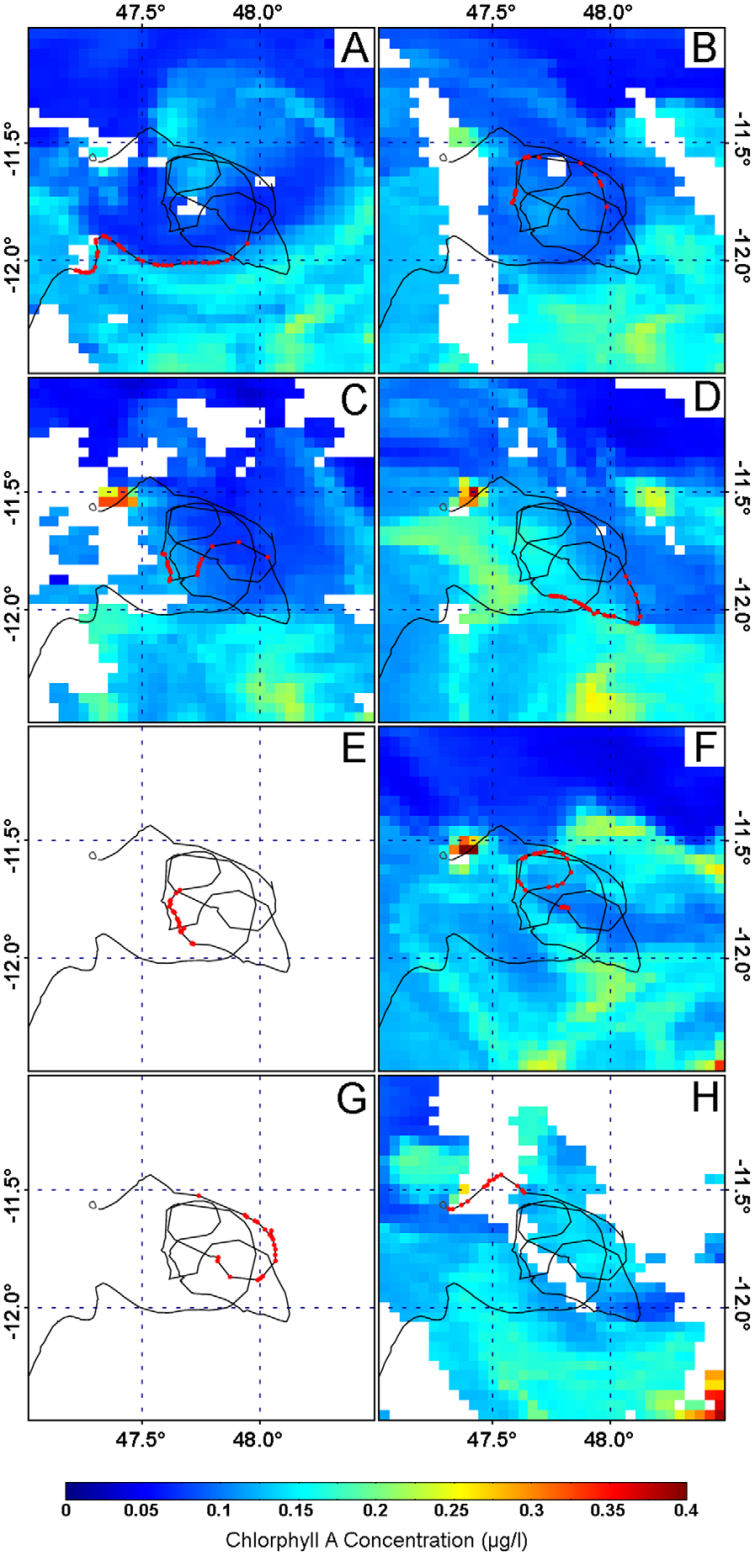
Daily maps of Chlorophyll A (ChA) concentration provided by MODIS-Aqua along with the last part of the homing path of turtle CO5, from 04 to 11 June 2009. The red dots represent the turtle' GPS locations recorded the same day as the map, and therefore illustrate the homing movements possibly impacted by submesoscale activity. ChA concentration is considered as a passive tracer advected by the ocean dynamics. Coherent meso- and submeso-scale structures are clearly observed in this temporal image sequence and the high ChA concentration represents the borders of these oceanographic structures. In these areas the norm of the current motion field is higher. White pixels represent areas where ChA concentration cannot be measured due to the cloud cover. Panel A: the path was following the external active border of a dipole mesoscale structure, composed of two eddies (not detectable as sea level anomalies because of theirs small sizes): the anticyclonic part is centered at 11.5°S–47.8°E and the cyclonic one at 11.7°S–47.4°E. Panel B: the dipole structure moved to south-east. The turtle was advected by the cyclonic eddy of the dipole. The displacement of the turtle followed the internal border of the cyclonic eddy. Panel C: the turtle reached the cyclonic structure to enter in a complex ocean dynamics configuration created by ejection of numerous energetic filaments by the principal dipole structure described in panel A. Panel D: ChA concentration increased and the different ejection filaments can be clearly represented. Panel E: no data available because of cloud cover. Panel F: the turtle turned around a submesoscale structure but the mixing of ChA is too high and gradient too low to have a clear detection of this submesoscale structure. Panels G & H: no ChA data available for the turtle's locations because of cloud cover.

As the spatial resolution of the current velocity field estimations are limited to 0.25° in both latitude and longitude (about 28 km), only mesoscale oceanic currents can be estimated, thus leaving out submesoscale currents. These smaller scale structures usually occur in the form of filaments or eddies with radii of a few kilometers lasting a few days. They may be very dynamic, involving locally strong currents, but are unpredictably distributed in both space and time [30]. Even if these submesoscale currents cannot yet be properly estimated, it is nevertheless possible to identify them by using Sea Surface Temperature (SST, obtained in the infra-red light spectrum) and Chlorophyll A (ChA, obtained in the visible light spectrum) daily data from MODIS-Aqua aboard EOS-PM satellite (http://modis.gsfc.nasa.gov/). To this aim, we built up ChA- and SST-based singular exponents maps, which provide a clear view of local turbulent motion [31], [32], using Yahia and Turiel's “FluidExponents”® software [33]. Because parts of the homing paths possibly disturbed by these submesoscale currents could not be properly corrected, they were removed from analysis (see an example in Fig. 6).

### Homing path analyses

Motor paths were rediscrestized with a 5-km step length and represented in a home-based frame of reference, with the *X* axis corresponding to the home direction (see [12] for details). Changes in abscissa (Δ*X* = 5*cos(θ−γ), where θ and γ stand for the local movement and the home directions, respectively), thus directly correspond to the homeward component, i.e. the extent to which a turtle moves towards (positive value) or away from (negative value) home at each step. For convenience, the release point coordinates were set to *X*_0_ = 0 and *Y*_0_ = 0. The homing paths of the 20 turtles that showed a strong motivation to home were first analyzed globally. For this purpose, we computed the homing efficiency of each turtle as the mean cosine of directional errors (θ−γ), which is equivalent to the straightness index (the ratio beeline distance/path length travelled; [34]).

Afterwards, we split the homing paths in three phases – initial, central and final – to perform separate analyses for each of them. The initial phase was defined as the phase starting at the release site and ending when the *X* coordinate of the motor path (i.e. the motor homeward component) became definitively positive. For turtles that initially and consistently swam in a correct direction (home direction ±90°) the *X* coordinate was always positive, and the initial phase was therefore reduced to zero. For turtles that initially swam in a wrong direction (opposite home direction ±90°) for a while before taking the correct one, the X coordinate of the motor was first more and more negative, but started to become less and less negative as soon as the turtle took a correct direction and finally became definitively positive. Some other turtles, however, tended to perform loops around the release point, as other displaced animals often do (e.g. [35]). In this case, the X coordinate of the motor path was alternatively positive and negative until the turtle stopped its looping behavior and started to home. Because of this potential looping behavior, the first occurrence of a positive X value does not necessarily indicate the end of the initial phase, which can be estimated to end when the X value became definitively positive. The final phase was defined as the phase starting when a turtle came for the first time within 50 km of home and ending when it entered the lagoon surrounding the home island (to filter out the very final, pinpointing stage of the homing journey, assumed to involve a fully different navigation process that operates only at small spatial scale) or abandoned homing. We acknowledge that this 50 km threshold is somewhat arbitrary. Given the results of our previous studies [5], [10]–[13], a radius of a few dozens of kilometers seems to be a suitable choice for looking at a navigation process working at medium scale. Globally similar results were obtained with other radii within the same order of magnitude, suggesting that this order of magnitude corresponds to the circle of confusion of the pelagic navigation mechanism. The central phase, which corresponds to the main pelagic phase, was defined simply as the intermediate phase occurring between the initial and final phases.

Each of these three phases required to be analyzed in a specific way. Animal movements are indeed best considered as biased correlated random walks, whose shape is determined by three main factors: goal attractiveness (directional bias), movement persistence (directional correlation, i.e. the tendency to keep the current moving direction for a while) and randomness degree [36], [37]. A strong movement persistence is extremely useful in enabling an animal to navigate quite efficiently even when it has to rely on noisy gradient fields [38], but can in turn be somewhat costly during the initial or final phase of a homing path. During the initial phase (at the release site and soon afterwards), an animal may start to move in a direction that does not lead towards home. As movement persistence and goal attractiveness will work against each other in this case, their interplay will generate a loop which can be quite large, depending on the relative weights of the two factors. A similar situation may occur during the final homing phase: the interplay between the two factors will lead the animal to perform a loop each time it misses the goal [39]. In contrast, during the central phase, goal attractiveness and movement persistence tend to work in synergy as the animal tends to head towards the goal at this stage. The mean cosine of directional errors is the best means to measure the navigational efficiency in this case [34]. In contrast, this parameter is an inappropriate estimator of navigational efficiency when movement persistence and goal attractiveness work in opposite ways because, in this case, they are likely to generate movement loops and the mean cosine of directional errors tends to be close to zero regardless the number and the sizes of the loops. Consequently, the mean cosine of directional errors was used to estimate the navigational efficiency of the turtles during the central phase, but other estimators had to be used to assess the performances of turtles during the initial and final phases of their trips.

The performances during the initial phase were simply estimated as the number of 5-km steps involved. The larger the step number (i.e. the path length) was, the greater difficulties a turtle experienced to quickly take the correct home direction after release. To estimate the difficulty of turtles to localize their nesting site during the final phase of their homing movement, we computed the mean square distance between turtles' successive locations and the goal location once they came within 50 km of home. This method provides reliable results in standard cases (e.g. [40]) but applying it directly to an animal that may have been drifted by currents may introduce some biases, as changes in distance can be due to the currents as well as to the turtle's own moving behavior. Potentially, this may have led to a lowering of statistical power through an increase of variance of the distribution of squared distances. To overcome this problem, we computed the mean squared distance based on serially equidistant (5 km) turtle locations along the motor paths instead of the ground-related paths. This approach is not perfect, but we could not identify a more sensible means to assess turtle performances in this particular case.
